# Phase Angle, Skeletal Muscle Function, and Molecular Phenotype in HFpEF: A Subanalysis of the SAUNA-HFpEF Study

**DOI:** 10.3390/nu18213483

**Published:** 2026-10-23

**Authors:** Ammar Abo Hwach, Aysenur Dasdemir, Doerte Ahrens, Matthias Roeper, Ivan Tanev, Michael Hansen, Mariam Janashia, Thomas Rauwolf, Mihaela Roxana Popescu, Manan Pareek, Alexander Schmeisser, Ruediger C. Braun-Dullaeus, Tarek Bekfani

**Affiliations:** 1 Department of Internal Medicine, Division of Cardiology and Angiology, University Hospital Magdeburg, 39120 Magdeburg, Germany; 2 Department of Cardiology, Elias Emergency University Hospital, Carol Davila University of Medicine and Pharmacy, 050474 Bucharest, Romania; 3 Center for Translational Cardiology and Pragmatic Randomized Trials, Department of Biomedical Sciences, Faculty of Health and Medical Sciences, University of Copenhagen, 1165 Copenhagen, Denmark; 4 Department of Cardiology, Copenhagen University Hospital—Herlev and Gentofte, 2900 Hellerup, Denmark

**Keywords:** HFpEF, sauna, bioelectrical impedance analysis, phase angle, skeletal muscle

## Abstract

Background: The phase angle (PhA) derived from bioelectrical impedance analysis (BIA) is a non-invasive marker of cellular integrity that has been associated with nutritional status, functional status and prognosis in heart failure (HF). Whether PhA is related to the skeletal muscle molecular phenotype in HF with preserved ejection fraction (HFpEF) remains unknown. Methods: In this exploratory subanalysis of the SAUNA-HFpEF trial, 17 patients with HFpEF underwent BIA with assessment of body composition, cardiopulmonary exercise testing, isokinetic assessment of quadriceps muscle strength, and measurement of circulating growth differentiation factor-15 (GDF-15), as well as nutrition-related and metabolic laboratory parameters before and after a 10-week sauna intervention. Paired vastus lateralis biopsies (*n* = 12) were analyzed by quantitative real-time polymerase chain reaction (qRT-PCR) for catabolic (FBXO32, GSK-3β), fatty acid metabolism (ACADL, CPT1B, CD36), and anabolic (mTOR, IGF-1) markers. Associations with PhA were assessed using Spearman correlation and repeated-measures correlation (rmcorr), with false discovery rate (FDR) correction for multiple testing. Results: Cross-sectionally, lower baseline PhA correlated with higher GDF-15 (r = −0.758; 95% CI −0.936 to −0.412), higher extracellular water to total body water (ECW/TBW) ratio (r = −0.744; 95% CI −0.941 to −0.350), higher expression of GSK-3β, ACADL, FBXO32, CD36, and CPT1B (all r ≤ −0.620), and lower isokinetic quadriceps strength (r = 0.523) (all FDR-adjusted *p* < 0.05). No significant associations were found for IGF-1, mTOR, or muscle mass. An exploratory comparison using a previously published PhA cut-off of 4.65° showed consistent differences. Longitudinally, PhA did not change significantly over the intervention, and longitudinal changes in PhA were unrelated to changes in molecular markers but correlated with changes in the ECW/TBW ratio (rmcorr r = −0.708; 95% CI −0.883 to −0.360). Body composition, waist circumference, the lipid profile, HbA1c and iron status were also assessed at both time points without any observed changes (all FDR-adjusted *p* > 0.05). Conclusions: In this small exploratory analysis, lower PhA was associated with a more catabolic and metabolically altered skeletal muscle gene expression profile in HFpEF. PhA may provide complementary, non-invasive information on muscle quality and cellular and fluid status. Its potential role in identifying patients who warrant a structured nutritional assessment deserves further investigation. These hypothesis-generating findings require validation in larger prospective studies.

## 1. Introduction

Heart failure with preserved ejection fraction (HFpEF) currently accounts for approximately 50% of all heart failure (HF) diagnoses [1] and is characterized by exercise intolerance that severely limits quality of life [2,3]. While the pathophysiology of HFpEF has traditionally focused on cardiac mechanisms [1,4], growing evidence highlights the critical role of peripheral skeletal muscle dysfunction as a major factor in reduced exercise capacity [5,6,7]. Patients with HFpEF show impaired skeletal muscle function, predominant muscle atrophy, altered fatty acid and glucose metabolism, and mitochondrial abnormalities compared to healthy controls and patients with heart failure with reduced ejection fraction (HFrEF) [5,6,8,9,10]. Circulating biomarkers of catabolism and inflammation, including growth differentiation factor-15 (GDF-15), have been identified as independent predictors of reduced muscle function in patients with HFpEF [9].

Although skeletal muscle alterations are recognized as important in HFpEF, an exact assessment of these changes usually requires invasive procedures such as muscle biopsies [6,8,9]. The phase angle (PhA), derived from bioelectrical impedance analysis (BIA) at 50 kHz, has emerged as a non-invasive, bedside-available parameter reflecting cellular integrity, body cell mass, and the distribution between intracellular and extracellular water compartments [11,12,13,14]. In BIA, a weak alternating current is passed through the body, and the measured impedance is separated into resistance (R), the opposition of intra- and extracellular fluids to current flow, and reactance (Xc), the capacitive effect of cell membranes. PhA is calculated as PhA (°) = arctan (Xc/R) × 180/π. Higher values indicate a larger body cell mass with intact cell membranes, whereas lower values indicate impaired membrane integrity, reduced cell mass or a shift in fluid from the intracellular to the extracellular compartment [11,13]. Beyond the phase angle, BIA-derived body water compartments have proven informative in HF, for example in detecting preclinical congestion and in relation to muscle strength and exercise capacity [15]. In the context of HF, lower PhA values have been consistently associated with worse functional status, higher *N*-terminal pro-B-type natriuretic peptide (NT-proBNP) concentrations and increased mortality risk [16,17,18,19,20,21]. A recent meta-analysis of seven studies comprising 1070 patients identified a PhA of 4.65° as the optimal cut-off for predicting increased mortality in HF populations consisting predominantly of patients with HFrEF (hazard ratio [HR] 2.10; *p* = 0.002) [19]. Furthermore, PhA has been shown to independently predict cardiorespiratory fitness in patients with obesity and HFpEF, even after adjusting for disease severity [22].

However, the relationship between PhA and skeletal muscle function and molecular pathology, including markers of protein degradation, fatty acid metabolism, and anabolic signaling, has not been investigated in HFpEF. It remains unclear whether PhA reflects the specific molecular muscle phenotype beyond its known associations with functional capacity and fluid status. Moreover, it is unclear whether changes in PhA following an intervention such as sauna correlate with changes in skeletal muscle molecular markers or clinical outcomes in HFpEF.

The SAUNA-HFpEF study was the first prospective mechanistic pilot study to demonstrate that supervised sauna bathing improved exercise capacity, skeletal muscle function and quality of life in patients with HFpEF. Additionally, the muscle biopsies revealed an anabolic upregulation and catabolic downregulation [23]. The dataset of the SAUNA-HFpEF study includes serial BIA measurements, which provides a novel opportunity to explore the relationship between PhA and skeletal muscle molecular biology in HFpEF.

The aim of this exploratory subanalysis was therefore twofold: (1) to investigate cross-sectional associations between PhA and skeletal muscle molecular markers, circulating biomarkers, body composition and functional capacity at baseline; (2) to evaluate whether changes in PhA following sauna intervention correlate with changes in these parameters.

## 2. Methods

### 2.1. Study Design and Population

This is a subanalysis of the SAUNA-HFpEF study, a prospective, single-arm, mechanistic pilot study investigating the effects of supervised sauna bathing on exercise capacity and skeletal muscle function in stable ambulatory patients with HFpEF [23]. The study design, inclusion and exclusion criteria, and intervention protocol have been described in detail previously [23,24]. In summary, patients with confirmed HFpEF (left ventricular ejection fraction ≥ 50%, evidence of diastolic dysfunction, and symptoms of HF) underwent a 10-week supervised sauna intervention (twice weekly, 60 °C, progressively increasing from 8 to 15 min per session). Assessments were performed at baseline and at the end of the intervention period.

All participants provided written informed consent. The protocol (No. 61/23) was approved by the Ethics Committee of Otto-von-Guericke University Magdeburg and complied with Good Clinical Practice and the Declaration of Helsinki [25]. The study is registered at ClinicalTrials.gov with the number NCT06978868. Detailed inclusion and exclusion criteria are presented in the design paper [24].

### 2.2. Sauna Intervention and Physical Activity

Patients attended two supervised sauna sessions per week at 60 °C for 10 weeks. Sessions were held in groups of up to five patients and were accompanied by study physicians. Sessions started at 8 min and were extended by 2 min every two weeks to 12 min; in weeks 7–10, they lasted 12–15 min. After each session, patients showered with water of moderate temperature and rested for 20 min. Blood pressure and heart rate were measured before each session, immediately afterwards and at the end of the rest period [24]. The adherence in the parent study was 97.2 ± 4.3%, and no adverse events occurred [23]. Physical activity was assessed by accelerometry (ActiGraph wGT3X-BT; ActiGraph LLC, Pensacola, FL, USA) as part of the parent study; step count, metabolic equivalents, sedentary time and time spent in moderate or vigorous activity did not change significantly during the intervention [23].

### 2.3. Bioelectrical Impedance Analysis and Phase Angle

Body composition was assessed by bioelectrical impedance analysis (BIA) using a validated multifrequency device (InBody 970; InBody Co., Ltd., Seoul, Republic of Korea). Measurements were performed in the morning after an overnight fast, at the time of blood sampling, in the standing position using eight-point tactile electrodes, after voiding, after a 15 min rest period and without prior physical exercise. Patients took their regular medication, including diuretics in those with an established indication, as prescribed. One patient with a cardiac pacemaker could not undergo BIA and was excluded from this subanalysis. The phase angle (PhA) at 50 kHz was calculated by the device from whole-body resistance and reactance at each measurement. The extracellular water to total body water (ECW/TBW) ratio was derived from segmental impedance measurements as a marker of fluid distribution. Skeletal muscle mass was estimated using validated BIA-derived equations.

### 2.4. Body Composition and Nutrition-Related Parameters

In addition to the phase angle, the following device-derived parameters of body composition were recorded at each measurement: body fat percentage, fat mass, estimated visceral fat area, fat-free mass index (FFMI), skeletal muscle index, protein mass and estimated basal metabolic rate.

### 2.5. Skeletal Muscle Biopsies and Molecular Analyses

Percutaneous skeletal muscle biopsies of the vastus lateralis were obtained at baseline and at the end of study, as described in the parent study [23]. Gene expression of molecular markers reflecting catabolic pathways (F-box only protein 32 [FBXO32/Atrogin-1], glycogen synthase kinase-3β [GSK-3β]), fatty acid metabolism (long-chain acyl-CoA dehydrogenase [ACADL], carnitine palmitoyltransferase 1B [CPT1B], fatty acid translocase [CD36]), and anabolic signaling (mechanistic target of rapamycin [mTOR], insulin-like growth factor-1 [IGF-1]) was quantified by quantitative real-time polymerase chain reaction (qRT-PCR) and expressed relative to the housekeeping gene glyceraldehyde-3-phosphate dehydrogenase (GAPDH) using the 2^−ΔΔCT^ method. Total RNA was isolated with TRIzol (Invitrogen, Carlsbad, CA, USA) after homogenization with a TissueLyser (Qiagen, Hilden, Germany); cDNA was synthesized with the RevertAid First Strand cDNA Synthesis Kit (Thermo Fisher Scientific, Waltham, MA, USA), and qRT-PCR was performed with Power SYBR Green PCR Master Mix on a StepOne Real-Time PCR System (Applied Biosystems, Foster City, CA, USA). Biopsies were available in a subset of patients (*n* = 12).

### 2.6. Functional Assessments and Circulating Biomarkers

Cardiopulmonary exercise testing (AMEDTEC ECGpro, version 5.10.007; AMEDTEC Medizintechnik Aue GmbH, Aue, Germany) was performed in the upright position on an electronically braked cycle ergometer using a 15 W/min ramp protocol, combined with spirometry with breath-by-breath gas exchange analysis (Omnia, version 2.2; COSMED S.r.l., Rome, Italy) and electrocardiography (CARDIOVIT AT-102 G2; SCHILLER AG, Baar, Switzerland), to determine peak oxygen consumption (peak VO_2_, mL/min/kg). Isokinetic quadriceps strength of the right quadriceps was assessed in the concentric mode using a Biodex dynamometer (Biodex System 4 Pro, Biodex Medical Systems Inc., Shirley, NY, USA) and expressed as maximal torque extension at 60°/s (Nm). Circulating growth differentiation factor-15 (GDF-15, pg/mL) was measured from venous blood samples at baseline and end of study using a commercially available enzyme-linked immunosorbent assay (ELISA; DuoSet ELISA DY957, R&D Systems, Minneapolis, MN, USA). In addition, routine nutrition-related and metabolic laboratory parameters were obtained from venous blood samples at both time points and analyzed in the central laboratory of University Hospital Magdeburg: total cholesterol, low-density lipoprotein (LDL) cholesterol, high-density lipoprotein (HDL) cholesterol, triglycerides, glycated haemoglobin (HbA1c), ferritin, transferrin saturation, thyroid-stimulating hormone (TSH) and serum electrolytes. Blood was drawn under fasting conditions.

### 2.7. Statistical Analysis

Continuous variables are presented as median [25%; 75%] or mean ± SD, categorical as *n* (%).

The primary analysis addressed cross-sectional associations between PhA and baseline values of all study parameters, using Spearman rank correlation coefficients (r) with 95% confidence intervals (CIs) obtained by non-parametric percentile bootstrapping. Longitudinal within-subject associations between phase angle and study parameters were assessed using repeated-measures correlation (rmcorr), which estimates the within-subject association between two repeatedly measured variables while accounting for between-subject differences in overall level [26]. The 95% CIs for repeated-measures correlations were derived using Fisher’s z transformation, as implemented in the rmcorr package.

Changes between baseline and the end of the study were assessed using the Wilcoxon signed-rank test for continuous variables and the McNemar test for paired categorical variables.

In an additional exploratory analysis, patients were dichotomized into low and high PhA groups using a cut-off of 4.65°, which a recent meta-analysis identified as the optimal predictor of increased mortality in HF populations consisting predominantly of patients with HFrEF (HR 2.10; *p* = 0.002) [19]. As this cut-off has not been validated in HFpEF or for skeletal muscle outcomes, and the resulting subgroups were very small, this analysis was considered exploratory and interpreted only in conjunction with the continuous analyses. Baseline differences in skeletal muscle gene expression and circulating GDF-15 between the groups were compared using the Wilcoxon rank-sum test and reported as Hodges-Lehmann estimates of the median difference with 95% CIs.

All *p*-values within each table were adjusted for multiple testing using the Benjamini–Hochberg false discovery rate (FDR) method [27]. An FDR-adjusted *p*-value < 0.05 was considered statistically significant. CIs were not adjusted for multiple testing. Given the exploratory nature of this subanalysis, all findings are regarded as hypothesis-generating. Analyses were performed using R v4.6.1 (R Foundation for Statistical Computing, Vienna, Austria) and RStudio v2026.07.1+147 (Posit Software, PBC, Boston, MA, USA).

## 3. Results

### 3.1. Study Population

Between October 2023 and February 2024, 17 of the 18 patients from the SAUNA-HFpEF study were included in this subanalysis (one patient was excluded due to a cardiac pacemaker) [age: 67.9 ± 8.6, f/m: 10/7]. Paired skeletal muscle biopsies were available in a subset of 12 patients. Baseline characteristics and end-of-study assessments are presented in Table 1.

### 3.2. Longitudinal Analyses: Change in PhA and Within-Subject Associations

PhA did not change significantly from baseline to end of intervention (median 4.5° vs. 4.9°; *p*(FDR) = 0.782). Table 2 presents repeated-measures correlations (rmcorr) between PhA and study parameters across baseline and end-of-study assessments. The ECW/TBW ratio showed a significant negative within-subject association with PhA (r = −0.708; 95% CI −0.883 to −0.360; *p*(FDR) = 0.012). No other parameter reached statistical significance after correction for multiple testing (all *p*(FDR) ≥ 0.05).

### 3.3. Longitudinal Changes in Body Composition and Nutrition-Related Parameters

Body composition and nutrition-related laboratory parameters are presented in Table 3. These included the phase angle, waist circumference, indices of adiposity and muscle mass, the extracellular water to total body water ratio, the lipid profile, HbA1c, iron status, thyroid-stimulating hormone and serum electrolytes. None of these parameters changed significantly between baseline and the end of the study (all *p*(FDR) ≥ 0.05).

### 3.4. Cross-Sectional Associations Between Baseline PhA and Study Parameters

**Figure 1:**
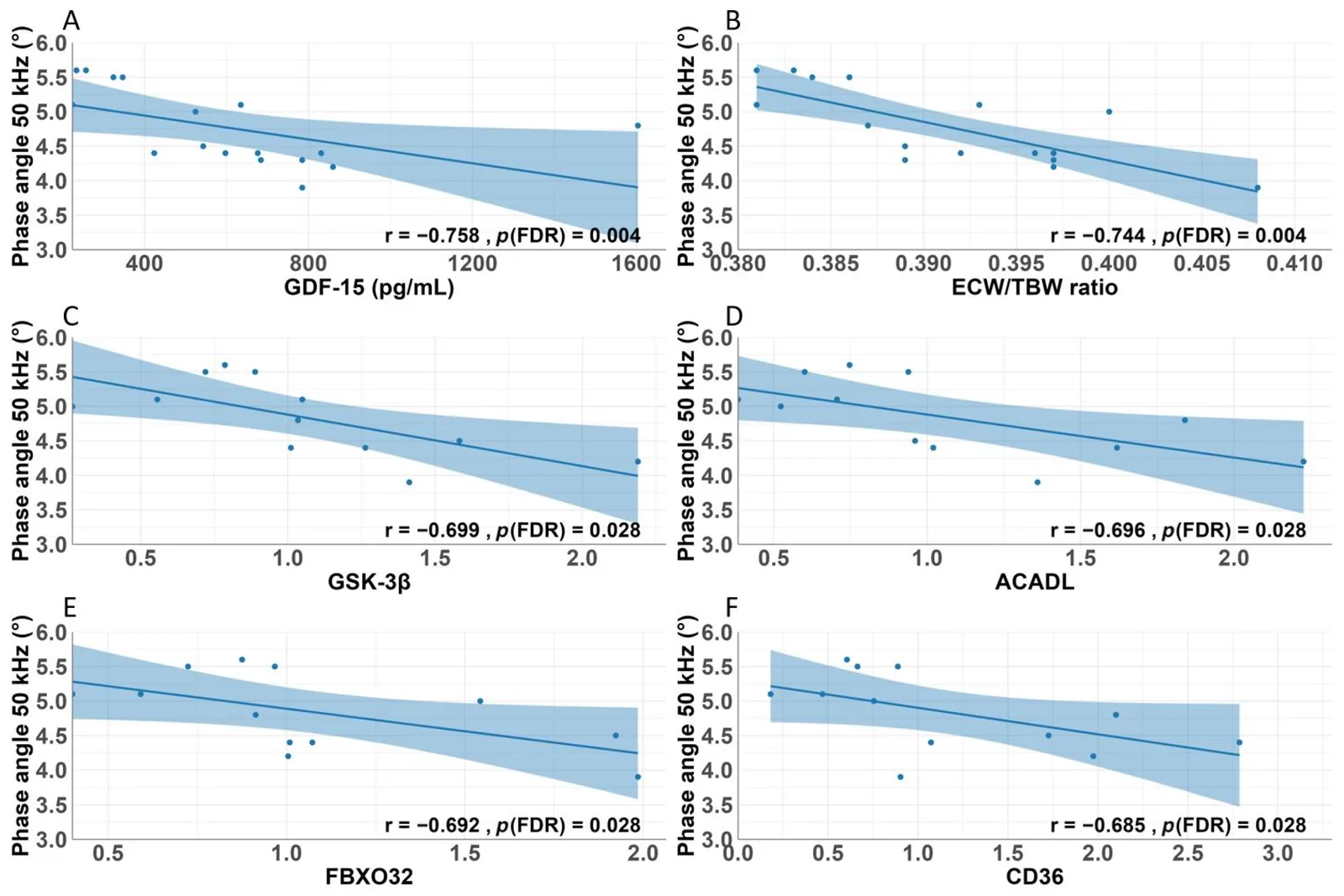
Cross-sectional Spearman rank correlations between the phase angle (PhA, 50 kHz) and molecular, metabolic, and fluid parameters at baseline in patients with heart failure with preserved ejection fraction (HFpEF). Scatterplots show the association between the phase angle and (**A**) circulating growth differentiation factor-15 (GDF-15), (**B**) the extracellular water to total body water (ECW/TBW) ratio, and the skeletal muscle gene expression of (**C**) glycogen synthase kinase-3β (GSK-3β), (**D**) long-chain acyl-CoA dehydrogenase (ACADL), (**E**) F-box only protein 32 (FBXO32/Atrogin-1), and (**F**) fatty acid translocase (CD36). Each point represents an individual patient; the solid line indicates the linear fit and the shaded area the 95% confidence interval (CI) band (shown for visualization only). Correlation coefficients (r) and false discovery rate (FDR)-adjusted *p*-values (*p*(FDR), Benjamini–Hochberg) are given within each panel.

Spearman rank correlation analyses between baseline PhA and baseline values of analyzed study parameters are presented in Table 4 and Figure 1. The strongest inverse correlations were observed for GDF-15 (r = −0.758; 95% CI −0.936 to −0.412; *p*(FDR) = 0.004) and the ECW/TBW ratio (r = −0.744; 95% CI −0.941 to −0.350; *p*(FDR) = 0.004).

Among skeletal muscle molecular markers, significant negative correlations were found for both catabolic markers [GSK-3β (r = −0.699; 95% CI −0.892 to −0.245; *p*(FDR) = 0.028), FBXO32 (r = −0.692; 95% CI −0.878 to −0.237; *p*(FDR) = 0.028)], as well as for all three markers of fatty acid transport and oxidation [ACADL (r = −0.696; 95% CI −0.885 to −0.258; *p*(FDR) = 0.028), CD36 (r = −0.685; 95% CI −0.876 to −0.256; *p*(FDR) = 0.028), and CPT1B (r = −0.622; 95% CI −0.911 to −0.104; *p*(FDR) = 0.047)]. No significant correlations were observed for the anabolic markers mTOR and IGF-1 or for skeletal muscle mass (all *p*(FDR) > 0.05). Maximal torque extension at 60°/s correlated positively with PhA (r = 0.523; 95% CI 0.126 to 0.789; *p*(FDR) = 0.047).

### 3.5. Exploratory Analysis Using the 4.65° Cut-Off

**Figure 2:**
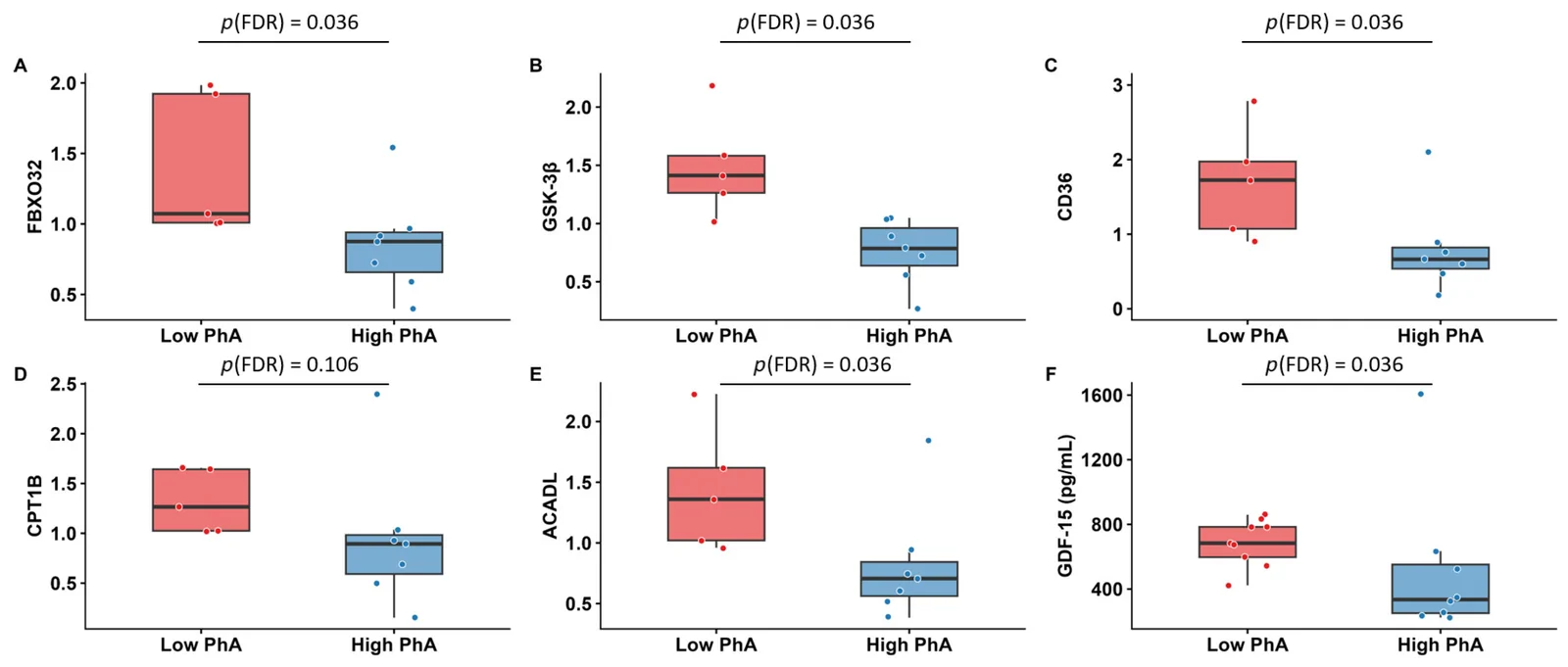
Baseline skeletal muscle gene expression and circulating growth differentiation factor-15 (GDF-15) in patients stratified by phase angle (PhA). In an exploratory analysis, patients were dichotomized at a previously published phase angle cut-off of 4.65° into a low-PhA (<4.65°, red) and a high-PhA (≥4.65°, blue) group. Boxplots show baseline levels of the catabolic genes (**A**) F-box only protein 32 (FBXO32/Atrogin-1) and (**B**) glycogen synthase kinase-3β (GSK-3β); the fatty acid transport and oxidation genes (**C**) fatty acid translocase (CD36), (**D**) carnitine palmitoyltransferase 1B (CPT1B), and (**E**) long-chain acyl-CoA dehydrogenase (ACADL); and (**F**) circulating GDF-15. Boxes represent the median and interquartile range, whiskers the range, and points individual patients. False discovery rate (FDR)-adjusted *p*-values (*p*(FDR), Benjamini–Hochberg, Mann–Whitney U test) are shown above each panel. Gene expression was assessed in skeletal muscle biopsies.

In an exploratory analysis of the baseline data, patients were dichotomized using the meta-analysis-derived cut-off of 4.65°. Patients with low baseline PhA (<4.65°) exhibited higher expression of catabolic and fatty acid metabolism markers and higher GDF-15 levels compared to patients with high baseline PhA (≥4.65°) (Table 5; Figure 2).

## 4. Discussion

This exploratory subanalysis of the SAUNA-HFpEF trial provides an initial indication that the phase angle, a non-invasive BIA-derived parameter, is associated with features of the skeletal muscle molecular phenotype in patients with HFpEF. In cross-sectional analyses, lower PhA was significantly associated with higher expression of catabolic markers (FBXO32, GSK-3β), upregulated fatty acid metabolism genes (ACADL, CPT1B, CD36), elevated circulating GDF-15, and reduced isokinetic quadriceps strength. These findings extend the known clinical associations of PhA. In contrast, median PhA increased only numerically during the intervention, from 4.5° to 4.9°, and thus above the previously defined cut-off of 4.65°; this change was not statistically significant and should not be overinterpreted. Within-subject changes in PhA were not significantly related to changes in molecular or functional markers.

### 4.1. PhA and Skeletal Muscle Molecular Markers

The cross-sectional inverse correlations between PhA and FBXO32 and GSK-3β are biologically plausible. FBXO32 regulates proteasomal degradation of muscle proteins [28] and is consistently upregulated in skeletal muscle of patients with HFpEF compared to both HFrEF and healthy controls [9]. GSK-3β plays a dual role in skeletal muscle homeostasis. On the one hand, it inhibits anabolic signaling through the mTOR pathway and promotes muscle protein breakdown [29]. On the other hand, GSK-3β is a key regulator of glycogen synthesis and insulin/IGF-1 signaling: in its active (non-phosphorylated) state, GSK-3β phosphorylates and inactivates glycogen synthase, thereby impairing glycogen storage and glucose metabolism [30,31]. The SAUNA-HFpEF study interpreted downregulation of GSK-3β following the sauna intervention as reflecting both reduced catabolic drive and improved metabolic signaling in skeletal muscle [23]. The present finding that lower PhA is associated with higher GSK-3β expression may therefore reflect both dimensions of this dysregulation: a more catabolic and metabolically altered muscle phenotype in patients with compromised cellular integrity. This second dimension may be of nutritional relevance. Skeletal muscle is the main site of postprandial glucose disposal, and impaired muscle glycogen synthesis is a hallmark of insulin resistance [32,33]. GSK-3β activity determines how efficiently ingested carbohydrate is stored as muscle glycogen [30,34]. Higher GSK-3β expression at a lower PhA might therefore indicate a reduced capacity of skeletal muscle to store dietary carbohydrate as glycogen, which would be clinically relevant in this cohort, in which 35% of patients had diabetes mellitus. However, GSK-3β activity and glucose handling were not assessed in the present study. Whether PhA is also related to the metabolic handling of nutrients within the muscle therefore remains a hypothesis for future studies.

The inverse associations between PhA and fatty acid metabolism genes (ACADL, CPT1B, CD36) add a further layer to this molecular characterization. Altered lipid metabolism is a hallmark of skeletal muscle dysfunction in HFpEF [5,9]. The upregulation of fatty acid transport and oxidation genes in patients with lower PhA may represent a compensatory response to mitochondrial inefficiency, as previously suggested in the context of HFpEF-related skeletal myopathy [5,9,35,36,37]. Alternatively, it may reflect a metabolic substrate shift in the setting of impaired glucose metabolism, a phenomenon described in skeletal muscle in patients with HF that is consistent with the concurrent GSK-3β upregulation observed in these patients [34,35,38]. We regard the second interpretation as at least as plausible: that this pattern reflects substrate oversupply rather than enhanced oxidative capacity. Notably, the sarcolemmal transporter CD36 was associated with PhA to a similar extent as the β-oxidation genes ACADL and CPT1B. Where CD36-mediated fatty acid uptake outpaces mitochondrial oxidative capacity, fatty acids are diverted toward lipid droplet storage and toward incompletely oxidized intermediates such as diacylglycerols, ceramides and acylcarnitines, which impair insulin signaling and promote mitochondrial stress and lipotoxicity [39].

A notable asymmetry emerged in the correlation pattern: while catabolic and metabolic markers showed strong associations with PhA, the anabolic markers mTOR and IGF-1 as well as skeletal muscle mass did not correlate significantly. This dissociation may have several explanations. First, the small sample size limits statistical power to detect moderate associations. Second, it is possible that PhA, which reflects cellular membrane integrity and the ratio of intracellular to extracellular water [40], is inherently more sensitive to catabolic processes that disrupt cellular integrity than to anabolic signaling, which may be preserved or variably activated as a compensatory mechanism even in the setting of net catabolism. Third, the lack of a significant correlation with skeletal muscle mass is consistent with the concept that PhA captures muscle quality rather than quantity [41,42], a distinction of particular relevance in HFpEF where sarcopenic obesity is prevalent and muscle mass alone may be a poor indicator of muscle health [22,43,44]. Our own data illustrate this. Conventional indicators of nutritional depletion were unremarkable in this cohort: body mass index was elevated, and both fat-free mass index and skeletal muscle mass were preserved and well above the thresholds used to define reduced muscle mass [45] (Table 3). A lower PhA, by contrast, was associated with a more catabolic and metabolically altered muscle gene expression profile. Assessment based on body weight or muscle mass alone might therefore not have captured these differences, suggesting that PhA may provide complementary information on muscle quality beyond these conventional measures [41,42]. Whether this reflects malnutrition could not be determined, as dietary intake and validated nutritional screening tools were not assessed.

The strongest cross-sectional correlation was observed between PhA and circulating GDF-15 (r = −0.758). GDF-15 is a stress-responsive cytokine increasingly linked to muscle wasting and cachexia in HF [46,47]. It drives catabolic pathways, suppresses appetite through glial cell line-derived neurotrophic factor family receptor α-like (GFRAL) signaling in the brainstem, and stimulates peripheral lipolysis [47,48]. In patients with HFpEF specifically, GDF-15 has been identified as an independent predictor of reduced muscle endurance [9,49,50].

The strong association between low PhA and high GDF-15, together with increased catabolic and metabolic muscle markers, suggests that PhA may be related to both systemic catabolic burden and local muscle alterations, which would make it a potentially useful non-invasive biomarker. From a nutritional perspective, a reduced PhA might therefore serve as a signal to prompt structured assessment of appetite, dietary intake and nutritional status in patients with HFpEF, none of which were captured in the present analysis. This hypothesis should be tested in studies that include a formal nutritional assessment, for example according to the Global Leadership Initiative on Malnutrition (GLIM) criteria [45].

In the exploratory analysis using the 4.65° cut-off, which was derived from a mortality meta-analysis in predominantly HFrEF populations [19], patients below this cut-off showed higher GDF-15 concentrations and higher expression of catabolic and fatty acid metabolism genes than those above it. These differences were directionally consistent with the continuous associations. However, given the very small subgroups and the wide confidence intervals, they do not establish that this cut-off identifies a distinct molecular phenotype in HFpEF. Whether a PhA cut-off is useful in HFpEF, and at which value, needs to be determined in larger cohorts.

### 4.2. PhA and Functional Capacity

PhA showed a significant positive correlation with isokinetic quadriceps strength, whereas the correlation with peak VO_2_ was not significant. This association should be examined in larger cohorts, given prior work demonstrating that PhA independently predicts cardiorespiratory fitness in patients with obesity and HFpEF after adjusting for disease severity [22].

### 4.3. Longitudinal Associations and Change in PhA

The only significant longitudinal association was between PhA and the ECW/TBW ratio, confirming the well-established biophysical relationship between these parameters [13,51,52]. PhA did not change significantly over the 10-week intervention, and no significant within-subject correlations were observed between changes in PhA and changes in molecular markers or functional parameters. This dissociation is notable given that the main published study demonstrated significant improvements in exercise capacity, muscle strength and molecular markers following the sauna intervention [23]. Several reasons may explain this finding. PhA may respond more slowly to therapeutic intervention than functional or molecular endpoints, requiring a longer intervention period or greater stimulus to produce detectable shifts in cellular integrity. Additionally, the small sample size limits the ability to detect modest longitudinal associations. Accordingly, the main findings of the present analysis are cross-sectional and do not allow conclusions relevant to causality.

## 5. Clinical Implications

These hypothesis-generating findings suggest that PhA might provide practical, non-invasive complementary information on skeletal muscle molecular status in patients with HFpEF. Because it only requires a standard BIA device, PhA can be assessed quickly and easily at the bedside. If validated in larger cohorts, PhA could help identify patients with more pronounced catabolic muscle dysfunction who might benefit from targeted interventions, such as exercise training or nutritional support. A low PhA could then prompt a structured nutritional assessment, for example according to the GLIM criteria [45], and, where indicated, a combined approach of adequate protein intake and supervised exercise, in line with recommendations on nutrition in HF [53]. This is particularly relevant in HFpEF, where caloric restriction improves exercise capacity but also reduces lean mass [54], so that interventions guided by body weight alone might risk aggravating the muscle deficit they are intended to address [46]. Whether PhA could also guide patient selection for emerging pharmacological strategies remains speculative and would require dedicated investigation in powered trials.

## 6. Limitations

Several limitations should be acknowledged. First, the small sample size (*n* = 17, with biopsies in *n* = 12) limits statistical power and increases the risk of both type I and type II errors, despite correction for multiple testing. Second, the main findings are cross-sectional, and the single-arm design of the parent study precludes causal inference regarding the relationship between PhA and intervention effects. Third, the PhA cut-off of 4.65° was derived from a mortality meta-analysis including predominantly HFrEF patients [19], has not been validated in HFpEF or for skeletal muscle outcomes, and resulted in very small subgroups; the analyses are therefore exploratory. Fourth, the molecular analyses were limited to gene expression; protein-level confirmation and functional assays would strengthen the biological interpretation. Fifth, nutritional status was not formally assessed: dietary intake, appetite and recent weight loss were not systematically recorded, and neither a validated nutritional screening instrument nor diagnostic criteria for malnutrition such as the GLIM criteria [45] were applied. Our findings therefore relate to muscle-related molecular and functional features rather than to nutritional status itself, and the nutritional implications discussed above remain hypothetical. Sixth, bioelectrical impedance-derived estimates are influenced by hydration status, which is particularly relevant in HF. Although BIA was performed under standardized conditions, diuretics were taken as prescribed (Table 1). An influence of fluid shifts on PhA and the ECW/TBW ratio, including their within-subject association, cannot be excluded. Seventh, the habitual physical activity, which may influence PhA, muscle strength and muscle metabolism, was not included in the present study. Although objectively measured physical activity did not change significantly during the intervention in the parent study [23], confounding by interindividual differences in physical activity cannot be excluded. Finally, the exploratory nature of this subanalysis and the multiple comparisons performed necessitate cautious interpretation, and the findings should be considered hypothesis-generating.

## 7. Conclusions

In this exploratory subanalysis of the SAUNA-HFpEF study, lower baseline PhA was associated with higher skeletal muscle expression of genes related to catabolism and fatty acid metabolism, higher circulating GDF-15, a higher ECW/TBW ratio and lower isokinetic quadriceps strength in patients with HFpEF. PhA may thus provide complementary, non-invasive information on muscle quality and cellular and fluid status that may not be captured by body weight or muscle mass alone. Within-subject changes in PhA during the 10-week sauna intervention were related to changes in the ECW/TBW ratio but not significantly to changes in molecular or functional markers. Whether a low PhA identifies patients who warrant a structured nutritional and functional assessment, and whether the 4.65° cut-off applies to HFpEF, should be tested in larger prospective studies that include formal nutritional assessment.

## Figures and Tables

**Table 1:** Baseline characteristics and end-of-study assessments.

Variable	Baseline	End of Study	*p*(FDR)
Demographic and clinical characteristics			
Age (years)	67.9 ± 8.6		-
Female/male (*n*)	10/7		-
NYHA II/III (%)	59/41	94/6	0.206
BMI (kg/m^2^)	30.0 [28.5; 33.0]	29.1 [26.6; 33.3]	0.480
Laboratory parameters			
NT-proBNP (pg/mL)	72.3 [47.4; 263.5]	140.0 [71.8; 214.0]	0.480
Hb (mmol/L)	8.40 [8.10; 8.75]	8.70 [8.35; 8.90]	0.235
Haematocrit	0.40 [0.38; 0.41]	0.42 [0.40; 0.43]	0.133
eGFR (mL/min)	82.2 [67.5; 85.8]	84.0 [69.3; 93.6]	0.367
Comorbidities			
Myocardial infarction (%)	17.6	17.6	-
Diabetes (%)	35.3	35.3	-
Hypertension (%)	88.2	88.2	-
AFIB (%)	23.5	23.5	-
Hypercholesterolaemia (%)	52.9	52.9	-
Medication			
Beta blocker (%)	82.4	82.4	-
ACE inhibitors and AT1-receptor-antagonists (%)	94.1	82.4	0.480
SGLT2-inhibitors (%)	56.2	68.8	0.480
Diuretics (%)	35.3	52.9	0.480
Aldosterone antagonists (%)	11.8	0.0	0.480

Continuous variables are presented as median [25%; 75%] or mean ± SD, categorical variables as %. Comparisons between baseline and end of study were performed using the Wilcoxon signed-rank test for continuous and the McNemar test for categorical variables, with *p*-values adjusted for multiple testing across the comparisons shown in this table using the Benjamini–Hochberg false discovery rate method. ACEi, angiotensin-converting enzyme inhibitor; ARB, angiotensin receptor blocker; ARNI, angiotensin receptor–neprilysin inhibitor; BMI, body mass index; eGFR, estimated glomerular filtration rate; FDR, false discovery rate; NT-proBNP, *N*-terminal pro-B-type natriuretic peptide; NYHA, New York Heart Association; SGLT2, sodium-glucose co-transporter 2.

**Table 2:** Repeated-measures correlations (rmcorr) between phase angle (50 kHz) and skeletal muscle gene expression, circulating GDF-15, fluid distribution, and functional capacity across baseline and end-of-study assessments.

Variable	r	95% CI	*p*(FDR)
ECW/TBW ratio	−0.708	[−0.883; −0.360]	**0.012**
GDF-15 (pg/mL)	0.299	[−0.195; 0.672]	0.547
FBXO32	0.015	[−0.540; 0.561]	0.961
GSK-3β	−0.429	[−0.793; 0.159]	0.506
ACADL	0.020	[−0.537; 0.565]	0.961
CPT1B	0.312	[−0.288; 0.737]	0.599
CD36	0.406	[−0.187; 0.782]	0.506
mTOR	0.109	[−0.470; 0.623]	0.961
IGF-1	0.526	[−0.036; 0.835]	0.389
Skeletal muscle mass (kg)	0.187	[−0.306; 0.602]	0.784
Maximal torque extension at 60°/s (Nm)	0.102	[−0.383; 0.543]	0.961
Peak VO_2_ (mL/min/kg)	−0.061	[−0.513; 0.418]	0.961

ACADL, long-chain acyl-CoA dehydrogenase; CD36, fatty acid translocase; CI, confidence interval; CPT1B, carnitine palmitoyltransferase 1B; ECW/TBW, extracellular water to total body water ratio; FBXO32, F-box only protein 32 (Atrogin-1); FDR, false discovery rate; GDF-15, growth differentiation factor-15; GSK-3β, glycogen synthase kinase-3β; IGF-1, insulin-like growth factor-1; mTOR, mechanistic target of rapamycin; peak VO_2_, peak oxygen consumption; r, repeated-measures correlation coefficient; rmcorr, repeated-measures correlation. The 95% CIs were derived analytically using Fisher’s z transformation. Significant *p*-values are highlighted in bold font.

**Table 3:** Body composition and nutrition-related laboratory parameters at baseline and end of study.

Variable	Baseline	End of Study	*p*(FDR)
Body composition			
Phase angle 50 kHz (°)	4.50 [4.40; 5.10]	4.90 [4.40; 5.40]	0.782
Waist circumference (cm)	99.4 [95.7; 117.7]	98.1 [93.3; 115.6]	0.261
Body fat (%)	37.9 [32.3; 41.0]	37.3 [32.6; 39.6]	0.906
Fat mass (kg)	29.4 [27.0; 36.7]	29.3 [25.1; 35.8]	0.733
Visceral fat area (cm^2^)	157.1 [122.1; 210.8]	152.8 [120.2; 190.0]	0.733
Fat-free mass index (kg/m^2^)	19.2 [17.4; 20.3]	19.4 [17.8; 19.9]	0.906
Skeletal muscle mass (kg)	28.4 [25.7; 34.6]	28.0 [24.7; 34.2]	0.733
Skeletal muscle index (kg/m^2^)	7.4 [7.0; 8.5]	7.9 [7.1; 8.5]	0.782
Protein mass (kg)	10.1 [9.2; 12.1]	10.0 [8.8; 12.0]	0.835
Basal metabolic rate (kcal/d)	1494 [1404; 1713]	1482 [1343; 1697]	0.835
ECW/TBW ratio	0.39 [0.39; 0.40]	0.39 [0.39; 0.40]	0.782
Nutrition-related and metabolic laboratory parameters			
Total cholesterol (mmol/L)	3.43 [3.14; 4.81]	3.40 [2.97; 4.31]	0.733
LDL cholesterol (mmol/L)	1.60 [1.46; 3.17]	1.54 [1.16; 3.11]	0.733
HDL cholesterol (mmol/L)	1.35 [1.07; 1.66]	1.32 [1.11; 1.66]	0.906
Triglycerides (mmol/L)	1.32 [0.68; 1.85]	1.08 [0.74; 2.02]	0.835
HbA1c (%)	6.31 [5.61; 6.61]	6.08 [5.67; 6.62]	0.906
Ferritin (ng/mL)	129.0 [108.5; 268.0]	126.0 [87.0; 189.0]	0.261
Transferrin saturation (%)	27.0 [22.5; 29.0]	25.0 [20.0; 25.5]	0.733
TSH (mU/L)	1.07 [0.55; 1.44]	1.24 [0.53; 1.50]	0.733
Sodium (mmol/L)	139.0 [138.5; 140.0]	140.0 [139.0; 140.5]	0.906
Potassium (mmol/L)	4.17 [4.08; 4.36]	4.03 [3.92; 4.25]	0.733

Values are presented as median [25%; 75%]. Body composition was assessed by multifrequency bioelectrical impedance analysis; waist circumference was measured with a tape measure. Comparisons were performed using the Wilcoxon signed-rank test, with *p*-values adjusted for multiple testing across the comparisons shown in this table using the Benjamini–Hochberg false discovery rate method. Numbers differ between variables because of incomplete paired laboratory sampling. ECW/TBW, extracellular water to total body water ratio; FDR, false discovery rate; HbA1c, glycated haemoglobin; HDL, high-density lipoprotein; LDL, low-density lipoprotein; TSH, thyroid-stimulating hormone.

**Table 4:** Baseline Spearman rank correlations between phase angle (50 kHz) and skeletal muscle gene expression, circulating GDF-15, fluid distribution, body composition, and functional capacity in patients with HFpEF.

Variable	r	95% CI	*p*(FDR)
GDF-15 (pg/mL)	−0.758	[−0.936; −0.412]	**0.004**
ECW/TBW ratio	−0.744	[−0.941; −0.350]	**0.004**
GSK-3β	−0.699	[−0.892; −0.245]	**0.028**
ACADL	−0.696	[−0.885; −0.258]	**0.028**
FBXO32	−0.692	[−0.878; −0.237]	**0.028**
CD36	−0.685	[−0.876; −0.256]	**0.028**
CPT1B	−0.622	[−0.911; −0.104]	**0.047**
Maximal torque extension at 60°/s (Nm)	0.523	[0.126; 0.789]	**0.047**
Peak VO_2_ (mL/min/kg)	0.502	[0.009; 0.811]	0.053
IGF-1	−0.165	[−0.650; 0.387]	0.730
Skeletal muscle mass (kg)	0.099	[−0.442; 0.659]	0.761
mTOR	−0.098	[−0.738; 0.541]	0.761

ACADL, long-chain acyl-CoA dehydrogenase; CD36, fatty acid translocase; CI, confidence interval; CPT1B, carnitine palmitoyltransferase 1B; ECW/TBW, extracellular water to total body water ratio; FBXO32, F-box only protein 32 (Atrogin-1); FDR, false discovery rate; GDF-15, growth differentiation factor-15; GSK-3β, glycogen synthase kinase-3β; HFpEF, heart failure with preserved ejection fraction; IGF-1, insulin-like growth factor-1; mTOR, mechanistic target of rapamycin; peak VO_2_, peak oxygen consumption; r, Spearman rank correlation coefficient. The 95% CIs were obtained by non-parametric percentile bootstrapping. Significant *p*-values are highlighted in bold font.

**Table 5:** Exploratory comparison of baseline skeletal muscle gene expression and circulating GDF-15 in patients below versus at/above 4.65°.

Variable	n (Low/High)	PhA < 4.65°	PhA ≥ 4.65°	Hodges-Lehmann Estimate [95% CI]	*p*(FDR)
Skeletal muscle gene expression (vastus lateralis biopsies, *n* = 12)					
GSK-3β	5/7	1.41	0.79	0.69 [0.23; 1.30]	**0.036**
FBXO32	5/7	1.07	0.88	0.42 [0.09; 1.20]	**0.036**
ACADL	5/7	1.36	0.71	0.61 [0.08; 1.29]	**0.036**
CD36	5/7	1.73	0.66	0.89 [0.15; 1.90]	**0.036**
CPT1B	5/7	1.27	0.89	0.53 [−0.02; 0.97]	0.106
Circulating biomarker (venous blood, *n* = 17)					
GDF-15 (pg/mL)	9/8	683.6	334.9	308.0 [49.0; 484.4]	**0.036**

Values are medians. Hodges–Lehmann estimates (low minus high PhA group) with 95% confidence intervals (CIs) are given in relative expression units for gene expression and in pg/mL for GDF-15; the Hodges–Lehmann estimate is the median of all pairwise differences between the two groups and therefore differs from the difference between the group medians. Gene expression was measured in vastus lateralis biopsies (*n* = 12; 5 vs. 7 patients) and GDF-15 in venous blood (*n* = 17; 9 vs. 8 patients). *p*-values were obtained with the Mann–Whitney U test and adjusted for multiple testing across the six comparisons shown using the Benjamini–Hochberg false discovery rate method. Owing to the small subgroups, this analysis is exploratory. ACADL, long-chain acyl-CoA dehydrogenase; CD36, fatty acid translocase; CPT1B, carnitine palmitoyltransferase 1B; FBXO32, F-box only protein 32 (Atrogin-1); FDR, false discovery rate; GDF-15, growth differentiation factor-15; GSK-3β, glycogen synthase kinase-3β; PhA, phase angle. Significant *p*-values are highlighted in bold font.

## Data Availability

Study data can be made available upon reasonable request to the corresponding author.

## Abbreviations

ACADL	long-chain acyl-CoA dehydrogenase
BIA	bioelectrical impedance analysis
BMI	body mass index
CD36	fatty acid translocase
CI	confidence interval
CPT1B	carnitine palmitoyltransferase 1B
ECW/TBW	extracellular water to total body water ratio
ELISA	enzyme-linked immunosorbent assay
FBXO32	F-box only protein 32 (Atrogin-1)
FDR	false discovery rate
GAPDH	glyceraldehyde-3-phosphate dehydrogenase
GDF-15	growth differentiation factor-15
GSK-3β	glycogen synthase kinase-3β
HF	heart failure
HFpEF	heart failure with preserved ejection fraction
HFrEF	heart failure with reduced ejection fraction
HR	hazard ratio
IGF-1	insulin-like growth factor-1
mTOR	mechanistic target of rapamycin
NT-proBNP	*N*-terminal pro-B-type natriuretic peptide
NYHA	New York Heart Association
peak VO_2_	peak oxygen consumption
PhA	phase angle
qRT-PCR	quantitative real-time polymerase chain reaction
rmcorr	repeated-measures correlation
